# *In situ* nanoarchitectonics of magnesium hydroxide particles for property regulation of carboxymethyl cellulose/poly(vinyl alcohol) aerogels

**DOI:** 10.1039/d1ra06556d

**Published:** 2021-11-01

**Authors:** Xiaohu Qiang, Xin Guo, Hongxi Su, Hong Zhao, Chengwei Ouyang, Dajian Huang

**Affiliations:** School of Material Science and Engineering, Lanzhou Jiaotong University Lanzhou 730070 PR China huangdj2015@yeah.net

## Abstract

Carboxymethyl cellulose (CMC)-based aerogels with low density, low thermal conductivity, and biodegradability are promising candidates for environmentally friendly heat-insulating materials. However, the application of CMC-based aerogels as insulation materials in building exterior walls is limited by the high water sensitivity, poor mechanical properties and high flammability of these aerogels. In this work, a simple hydration method was used to generate magnesium hydroxide (MH) directly from CMC/polyvinyl alcohol (PVA) mixed sol with active MgO obtained by calcined magnesite as the raw material. A series of composite aerogels with different MH contents were prepared through the freeze-drying method. Scanning electron microscopy showed that nanoflower-like MH was successfully synthesised *in situ* in the 3D porous polymer aerogel matrix. Compared with the mechanical properties and water resistance of the original CMC/PVA composite aerogels, those of the composite aerogels were significantly improved. In addition, the flame retardancy of the CMC/PVA composite aerogels was greatly enhanced by the introduction of MH into the polymer matrix, and the limiting oxygen index reached 35.5% when the MH loading was 60%.

## Abstract 2

Flame retardant efficiency of magnesium hydroxide in cellulose aerogels improved by *in situ* formation.

## Graphical abstract

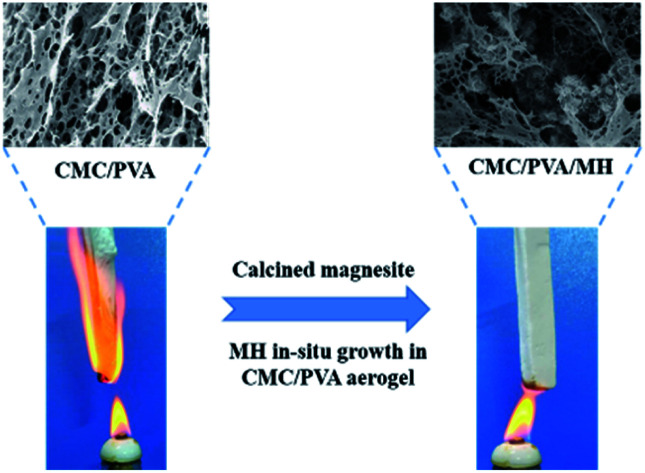

## 1. Introduction

Aerogels are 3D porous networks with low density, low thermal conductivity, and high specific surface area and porosity.^1–6^ Benefitting from these characteristics, aerogels have great potential for use in building energy-saving insulation and thermal pipelines and are considered alternatives to traditional thermal insulation materials, such as expanded polystyrene and rigid polyurethane foams.^7–9^ Carboxymethyl cellulose (CMC), which is derived from the carboxymethylation of hydroxyl groups on the cellulose backbone, is one of the most valuable and important derivatives of cellulose. It is widely sourced, non-toxic, biodegradable and soluble in water.^10–13^ CMC aerogels combine the advantages of cellulose and traditional aerogels and are promising candidate materials in thermal insulation.^11^ However, the mechanical properties and water sensitivity of pure CMC aerogels do not meet application requirements, and the flammability of these aerogels hinder their large-scale application and production.^13,14^

Magnesium hydroxide (MH) is one of the most widely used environmentally friendly fillers and flame retardants in the polymer industry.^15–17^ However, the high polarity and high specific surface area of MH lead to strong agglomeration between grains, resulting in serious defects at the interface of polymer materials.^16^ This compatibility problem caused by agglomeration affects the flame retardant efficiency of MH as a flame retardant and destroys the mechanical properties and processability of the matrix material to a large extent.^18–20^ Simple physical blending between an inorganic filler and the polymer matrix is unsatisfactory, especially in the case of high filler loading.^20,21^ Han *et al.*^22^ introduced MH nanoparticles into cellulose aerogels through the blending method. Scanning electron microscopy (SEM) images showed that the uniform porous structure collapsed due to the agglomeration of MH nanoparticles. Therefore, good dispersibility and stable interfacial adhesion strength between MH and the polymer matrix are needed for MH to execute an excellent filler function.^23^

To solve this problem effectively, most researchers have focused on the modification of MH by using silane coupling agents,^18,24,25^ saturated or unsaturated high fatty acid salts,^23,26,27^ titanate coupling agents^28,29^ and other modifiers.^24,29^ The introduction of these modifiers improves the dispersion of MH to varying degrees, but it is accompanied with new process costs and environmental problems. The *in situ* generation method directly forms a filler in the matrix to improve the dispersion and interfacial adhesion.^20,30,31^ Compared with the conventional methods of preparing MH, such as the sol–gel technique, precipitation of magnesium salt with an alkaline solution, hydrothermal technique and ammonia gas bubbling reactors,^32–36^ the method of *in situ* hydration generation is simpler, less expensive and more environment friendly.^35^ MH flame retardant can be prepared through a one-step method, and the compatibility issue with the polymer aerogel matrix can be solved. However, the microscopic morphology of MH particles can affect their dispersion in the polymer matrix, which in turn influences the flame retardant efficiency. Flake-shaped MH has low surface energy and surface polarity, resulting in good dispersion in the polymer matrix and high flame retardant efficiency.^29,33,35–37^ In the preparation of MH *via* MgO hydration, the activity of MgO is a key factor that affects the degree of hydration and morphology of MH. Many studies have shown that the calcination of magnesite by controlling the appropriate calcination temperature and time is an effective method to obtain active MgO.^38–40^

In this work, polyvinyl alcohol (PVA) was introduced to compensate for the inadequate mechanical properties of pure CMC aerogel with strong bonding and ductility.^41,42^ A simple hydration method was used to generate MH directly from CMC/PVA mixed sol with the active MgO obtained by calcined magnesite as the raw material to improve the dispersion of MH in the CMC/PVA matrix. Then, a series of CMC/PVA/MH composite aerogels were prepared through the vacuum freeze-drying method, and their microstructure, mechanical properties and flame retardant properties were studied and analysed.

## 2. Experimental

### 2.1. Materials

Sodium carboxymethyl cellulose (CMC) and polyvinyl alcohol (PVA) were provided by Sinopharm Chemical Reagent Co., Ltd. (China), both of which were of analytical grade. Magnesite was procured from Liaoning victory fire retardant material technology Co., Ltd. The distilled water was used for the whole experiments.

### 2.2. Preparation of CP/MH aerogels

**Fig. 1:**
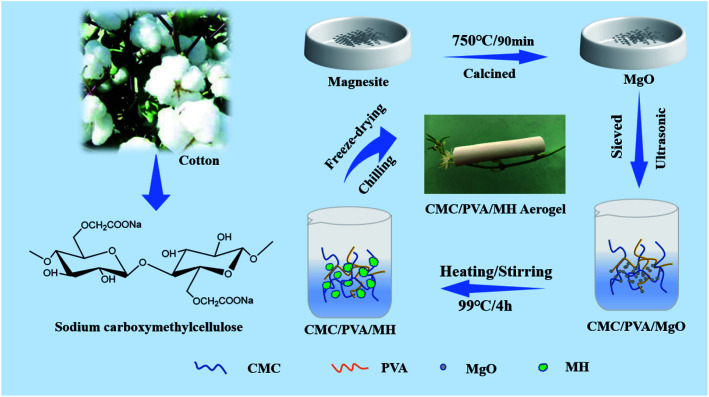
Preparation schematic of CP/MH composite aerogels.

The schematic of the preparation process for CP/MH composite aerogels is shown in Fig. 1. Firstly, a certain amount of magnesite is put in the crucible, drying water in the oven at 80 °C and then calcined in a high-temperature energy-saving chamber furnace. The calcination temperature is 750 °C and the calcination time is 90 min. Next, 2 g PVA was added into CMC solution (200 mL, 1.5 wt%), they were then heated and stirred in a water bath at 99 °C until PVA is completely dissolved and mixed well with CMC. After that, 0.86 g calcined magnesite was dispersed by ultrasonic for 20 min and added to the CMC/PVA mixed sol, continue to heat and stir for 4 h to make MgO hydrated to generate MH. Afterwards, the obtained mixed sol was poured into the mold and transferred to the refrigerator at −40 °C for 24 h. Finally, it was freeze-dried in a vacuum freeze dryer (−60 °C, 1 Pa) for 60 h to obtain CMC/PVA/MH composite aerogel with 20% theoretical load of MH, denote it as CP/MH20; in a similar way, three other groups of samples with MH content of 0%, 40%, and 60% were prepared, they were labeled as CP/MH0, CP/MH40 and CP/MH60, respectively. The composition ratio of several composite aerogels prepared in this study is shown in Table 1.

**Table 1:** Composition of various composite aerogels

Sample	CMC (g)	PVA (g)	MgO (g)
CMC/PVA-0% MH (CP/MH0)	3	2	0
CMC/PVA-20% MH (CP/MH20)	3	2	0.86
CMC/PVA-40% MH (CP/MH40)	3	2	2.29
CMC/PVA-60% MH (CP/MH60)	3	2	5.17

### 2.3. Characterization

A JEOL JSM 6510LV scanning electron microscopy (SEM) (JEOL, Japan) equipped with energy dispersive X-ray spectroscopy (EDS) at an acceleration voltage of 7 kV was used to observe the microstructure of aerogel samples, the surfaces of the samples were sputter-coated with a gold layer before examination.

X-ray diffraction (XRD) patterns were collected using an X'pert PRO X-ray power diffractometer equipped with a Cu-Kα radiation source (40 kV, 30 mA) (PAN analytical Co., Netherlands) from 5 to 80°, and the scan speed is 5° min^−1^.

Fourier transform infrared (FT-IR) spectra of composite aerogel samples were recorded on a FTIR spectrometry (Thermo Nicolet 6700, Thermo Fisher, USA) in the wavenumber range of 4000–400 cm^−1^.

The mechanical properties of samples were tested by AG-IS material testing machine (Shimadzu Co., Ltd., Japan). Before testing, the samples were cut into cylinders (12 mm in diameter and 10 mm in height) and placed in a dryer with saturated Mg (NO_3_)_2_ solution at the bottom for 24 h (50% humidity). The compression speed and compression distance are 1 mm min^−1^ and 6 mm, respectively. Five specimens at least were repeated to determine the average values in order to obtain reproducible results.

Evaluation of water resistance by measuring the moisture absorption (MU) of specimens in saturated NaCl. The samples were cut into small pieces and dried in an oven at 70 °C for eliminate moisture, and the initial mass *M*_0_ was recorded, then the mass *M*_1_ was weighed after 72 h storage in a desiccator with NaCl saturated salt solution (75% humidity) at the bottom. Five specimens at least were repeated to determine the average values in order to obtain reproducible results. In addition, we recorded the dissolving-expanding of aerogels after immersion in distilled water for 24 h. Moisture uptake was calculated as following equation:

1 MU = (*M*_1_ − *M*_0_)/*M*_0_

Thermogravimetric analysis (TGA) was performed from 30 °C to 600 °C at the increasing rate of 10 °C min^−1^ under a steady nitrogen flow of 40 mL min^−1^ on an STA409 thermal gravimetric analyzer (PerkinElmer Co., USA).

The limiting oxygen index (LOI) values were calculated by an HC-2C oxygen index meter (Jiangning, China) according to the standard method (GB/T 2406.2-2009), and all samples were prepared into a strip with the dimension of 100 mm × 15 mm × 5 mm.

## 3. Results and discussion

### 3.1. Morphology characteristics of composite aerogels

**Fig. 2:**
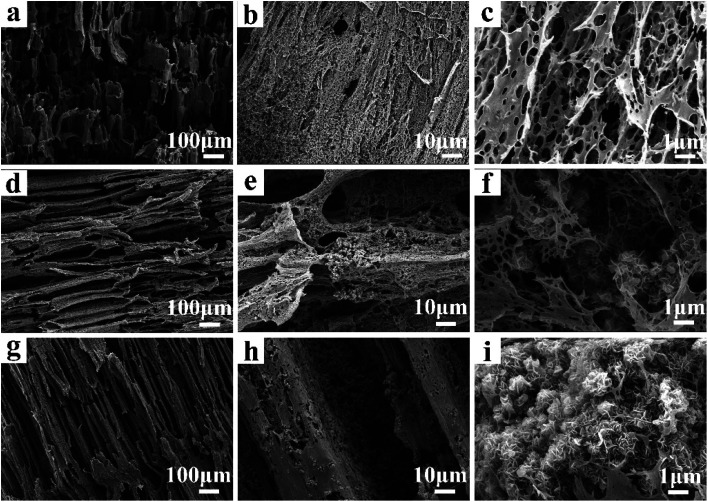
SEM images of composite aerogel (CP: a–c; CP/MH40: d–f; CP/MH60: g–i).

**Fig. 3:**
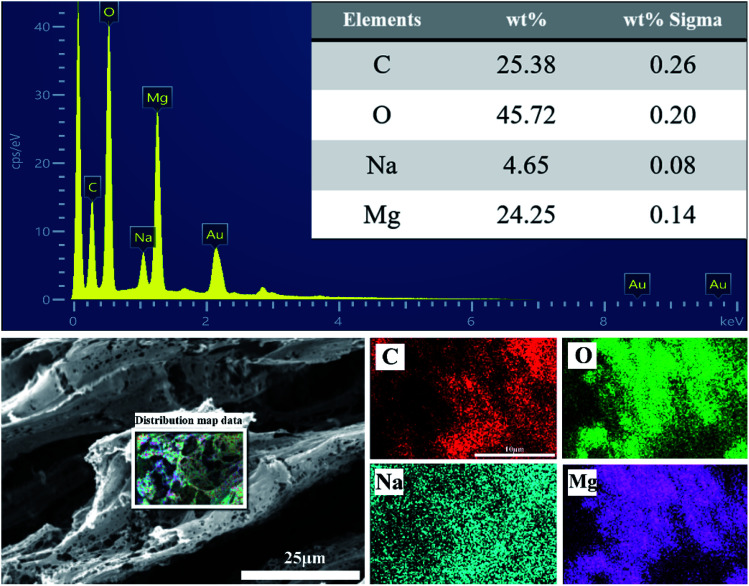
EDS elemental analysis of CP/MH60 composite aerogel.

The structure and morphology of CP aerogel and CP/MH composite aerogels were characterised through SEM and are shown in Fig. 2. The CP composite aerogel presented a 3D network with a hollow interior and extremely high porosity (Fig. 2a). After being loaded with MH, the CP/MH40 and CP/MH60 composite aerogels still maintained the layered porous structure of the cellulose aerogel; they had uniform voids, and no obvious collapse occurred (Fig. 2d–g). This result may be due to the *in situ* formation of MH, which improved the dispersion of MH in the polymer matrix and prevented the structural collapse caused by MH agglomeration. It may also be related to the high skeleton strength caused by the strong adhesion of PVA.^42^ High magnification showed that the network structure of the aerogel provided a good space for the *in situ* generation of MH. MH was embedded and semi-embedded in the pore network of the aerogel and supported the skeleton of the aerogel carrier (Fig. 2e and h). Meanwhile, the nano-sized flake MH with high specific surface area was clearly identified *via* high-magnification SEM and showed a spherical flower-like aggregate as a whole, which is similar to the observation of a previous study.^37,43^ This MH with a lamellar structure had low surface polarity and surface energy, which further enhanced the dispersion in the polymer.^29,37^ This result may be related to the high activity of MgO obtained after the calcination of magnesite, indicating that MH flame retardant was successfully grown *in situ* in the CMC/PVA composite matrix (Fig. 2f–i). In addition, energy-dispersive X-ray (EDS) elemental analysis was performed to determine the main elements in the CP/MH60 composite aerogel (Fig. 3). The results revealed that the main elements in the composite aerogel were C, O, Na and Mg. Mg had a high content, and the peak of Au was due to the gold spray layer on the surface of the sample.

### 3.2. X-ray diffraction

**Fig. 4:**
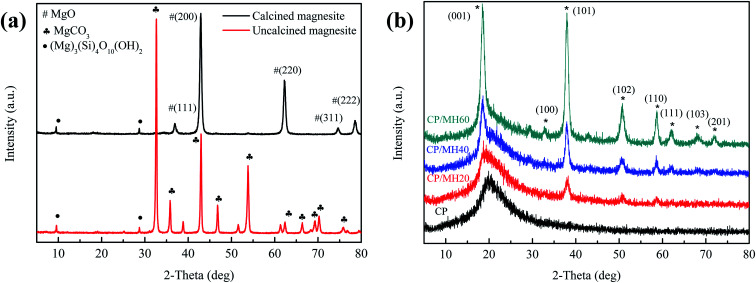
XRD patterns of (a) calcined magnesite and uncalcined magnesite (b) CP, CP/MH20, CP/MH40 and CP/MH60.

Fig. 4a shows the X-ray diffraction (XRD) pattern of the original magnesite and the magnesite after calcination (750 °C, 90 min). The results show that the XRD pattern of magnesite powder is mainly the characteristic diffraction peak of MgCO_3_. After calcination, the characteristic peaks of MgCO_3_ disappeared, and the diffraction peaks at 2*θ* = 36.9°, 42.92°, 62.32°, 74.66° and 78.82° corresponded to the (111), (200), (220), (311) and (222) crystal planes of MgO, respectively, this indicates that the main component of magnesite is MgCO_3_, and MgO is obtained by thermal decomposition at high temperature. In addition, the weak diffraction peaks at 2*θ* of 9.56° and 28.72° belong to the characteristic peaks of talc powder (Mg_3_[Si_4_O_10_] (OH)_2_).

Fig. 4b shows the X-ray diffraction (XRD) patterns of pristine CP aerogel and CP/MH composite aerogels with different MH contents. The diffraction peaks at about 2*θ* = 19.9° were ascribed to the diffraction of the CP matrix (Fig. 4a). After the introduction of MH, the diffraction peaks of CP/MH20, CP/MH40 and CP/MH60 at 2*θ* were 18.52°, 32.83°, 37.98°, 50.78°, 58.60°, 64.11°, 68.18° and 72.06°, which corresponded to the (001), (100), (101), (102), (110), (111), (103) and (201) crystal planes of MH, respectively.^29,44^ Amongst these peaks, the diffraction peak of CP/MH60 was the most obvious, indicating that MH was successfully generated *in situ* in the CP composite aerogel matrix (Fig. 4b–d). In addition, no obvious characteristic diffraction peaks of MgO were found, indicating a high degree of hydration. This result can be attributed to the MgO with high activity obtained by the calcination of magnesite.

### 3.3. Fourier transform infrared analysis

**Fig. 5:**
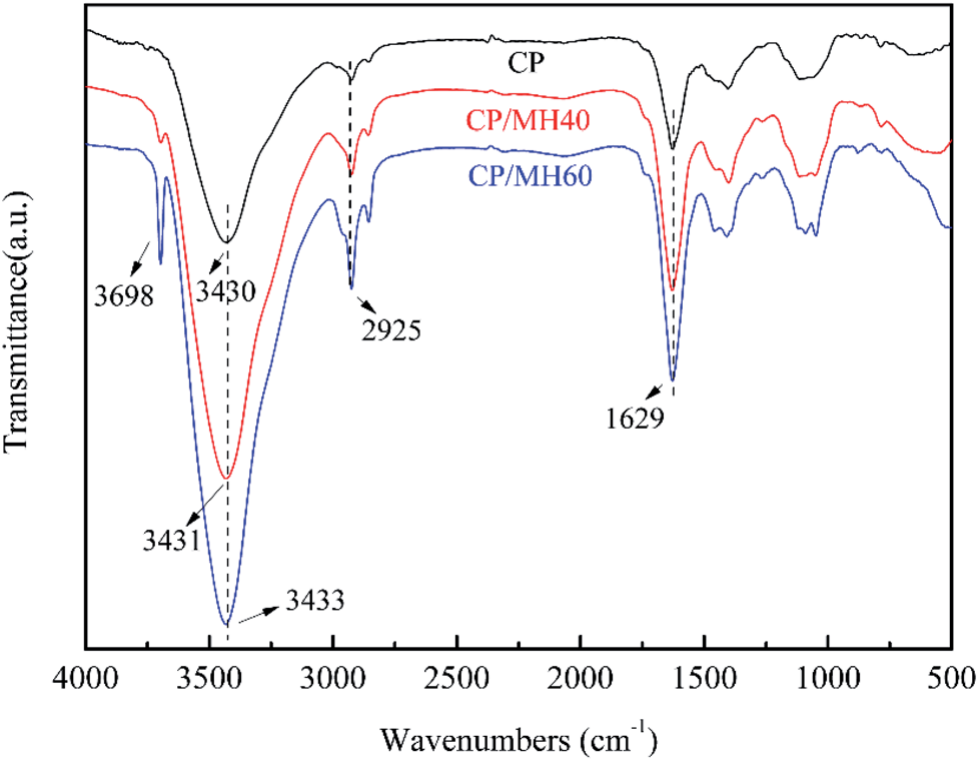
The FTIR spectra of CP, CP/MH40 and CP/MH60.

Fig. 5 shows the Fourier transform infrared (FTIR) spectra of CP, CP/MH40 and CP/MH60. The characteristic peak of CP at 3430 cm^−1^ was attributed to the O–H stretching vibration band. After introducing MH into the polymer matrix, the O–H stretching vibrational band was shifted compared to CP, from 3430 cm^−1^ to 3431 cm^−1^ and 3433 cm^−1^, respectively, which indicates the existence of hydrogen bonding interactions between CP and MH.^45^ In addition, the characteristic peaks of MH appeared at 3698 cm^−1^ in CP/MH40 and CP/MH60, indicating that MH flame retardant was successfully introduced into the CP aerogel matrix *via in situ* formation.^29,46,47^ The peak at 2925 cm^−1^ is attributed to the C–H stretching vibration of CH_2_ and CH_3_ groups. The adsorption band at 1629 cm^−1^ is due to the asymmetric stretching vibration of C=O, which is a typical adsorption band of carboxylate.^13^

### 3.4. Mechanical properties of composite aerogels

**Fig. 6:**
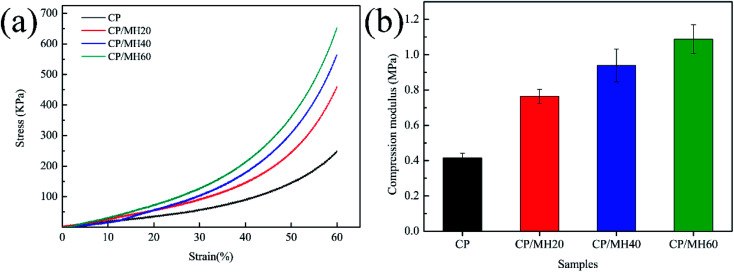
Compression stress–strain curves (a) and compression modulus (b) of CP and CP/MH composite aerogels.

Mechanical properties pose unavoidable problems in aerogel research.^22^ The compressive stress–strain curve (Fig. 6a) shows that the composite aerogel sample in this study exhibited the characteristics of plastic deformation under external force.^48^ The rate of curve rise was accelerated sequentially with increasing MH content. Correspondingly, the compressive modulus increased with the increment of MH content (Fig. 6b). For the pristine CP aerogel, the compressive modulus was only 0.42 MPa, and for the composite aerogel CP/MH60, the compressive modulus was 1.09 MPa (enhanced by 159.5%). This result may be attributed to the formation of MH in the CP aerogel matrix, which played a filling role by providing adequate force points for the porous aerogel matrix network and increasing the density of the composite aerogel.^49,50^ In addition, the hydrogen bonding interaction between CP and MH further enhanced the strength of the porous skeleton, making it highly capable of resisting collapse caused by external forces.

### 3.5. Water-resistance properties of composite aerogels

**Fig. 7:**
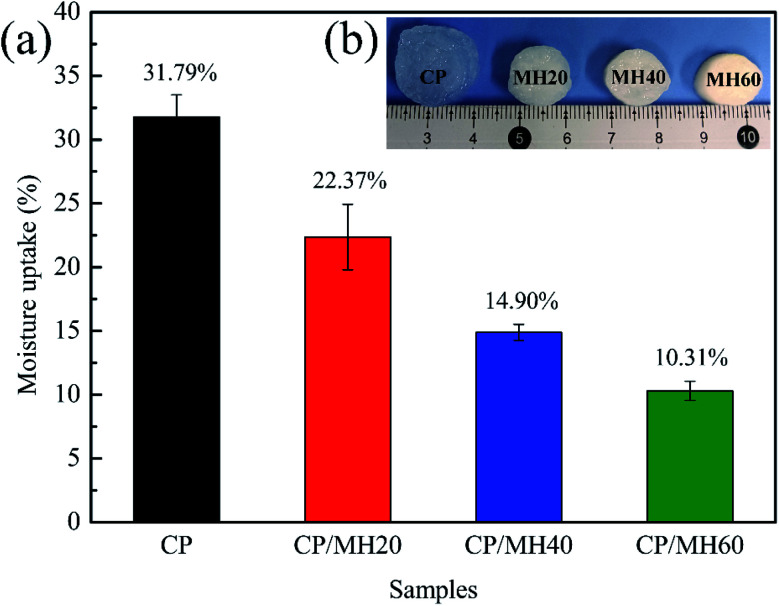
Water resistance properties of composite aerogels: (a) moisture uptake and (b) digital photographs after 24 h immersion in water.

Water resistance performance can be an important factor in defining the application of aerogel construction materials. Fig. 7a shows the moisture uptake (MU) rates of several sets of aerogel specimens in an environment with certain humidity. The MU value of pure CP aerogel is as high as 31.79% because of the high hydrophilicity of CMC and PVA due to the presence of a large number of hydrophilic groups.^41,51^ However, after introducing MH into the CP aerogel matrix by *in situ* generation method, the MU value of CP/MH underwent a significant decrease and decreased sequentially with the increase in MH content. Specifically, the MU value of CP/MH60 decreased to 10.31% (decreased by 67.59%). After soaking several aerogels in distilled water for 24 h (Fig. 7b), the neat CP aerogel underwent severe swelling, whereas the three other groups of samples with added MH maintained a relatively intact initial structure. This result is consistent with the results of the moisture uptake rate test. Similar to previous studies, Huang *et al.* used *in situ* cross-linking of sodium alginate by Ca^2+^ to reduce the swelling of agar aerogels in water.^52^ On the one hand, this result may be ascribed to the fact that the introduction of MH enhances the denseness of the polymer backbone and hinders the entry of water molecules; on the other hand, the hydrogen bond interaction between MH and CP matrix enhanced the stability of the composite aerogel.^51^

### 3.6. Thermal stability of composite aerogels

**Fig. 8:**
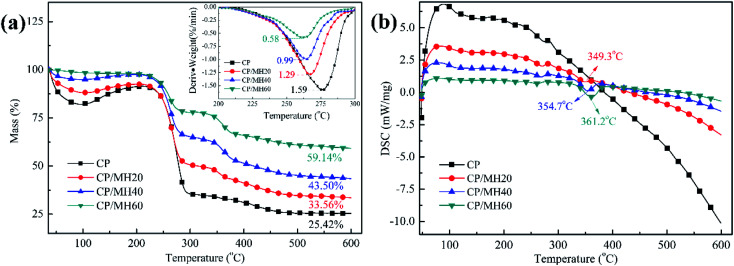
TGA weight loss (a) and DSC (b) curves of the various composite aerogels.

Thermal stability is crucial for the practical application of insulation materials. Thermogravimetric analysis (TGA) (Fig. 8a) and differential scanning calorimetry (DSC) (Fig. 8b) curves were utilised to analyse the thermal stability of the composite aerogels. The weight loss of several sets of aerogel samples below 200 °C was due to water volatilisation, and the main mass loss in the region of 200–300 °C was attributed to the pyrolysis of many hydroxyl groups in CMC and PVA. The differential thermogravimetry (DTG) curves at this stage show that the rate of thermal decomposition of the composite aerogels decreased significantly with the increase in MH content. The maximum thermal decomposition rate of the pure CP aerogel at this stage was 1.59%/min, whereas that of CP/MH60 decreased to 0.58%/min, indicating that MH enhanced the thermal stability of the composite aerogel (Fig. 8a). The secondary weight loss of the three CP/MH composite aerogels at 300–400 °C phase was due to the decomposition of MH. The DSC curve at this stage also demonstrates the improvement of the thermal stability of the composite aerogels with the increase in MH content (Fig. 8b). The final residue mass of the composite aerogels was significantly higher than that of the pure CP aerogel.

### 3.7. Flame retardant properties of the composite aerogels

**Fig. 9:**
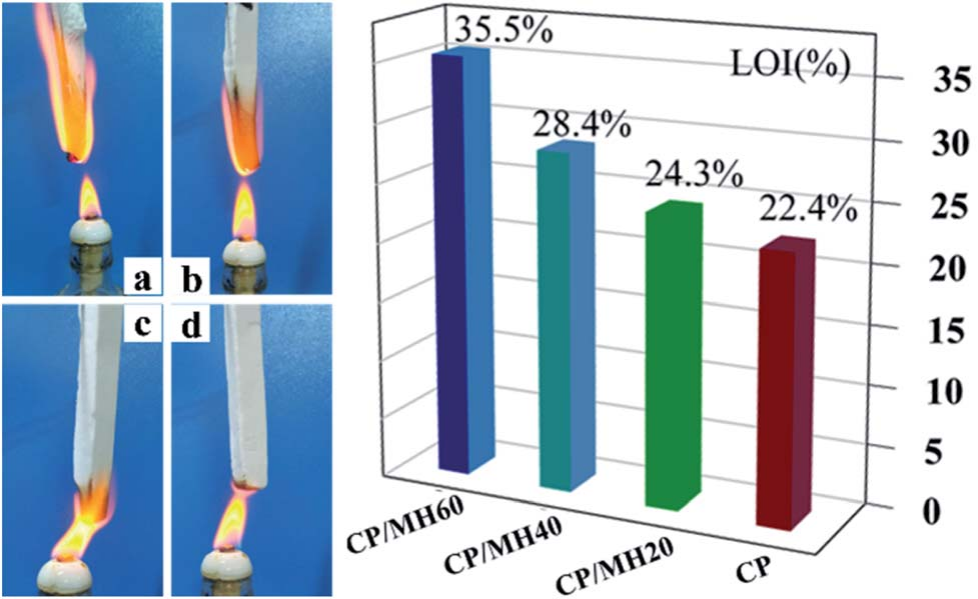
Optical photos of (a) CP, (b) CP/MH20, (c) CP/MH40 and (d) CP/MH60 composite aerogels in a combustion environment (3 s) and LOI of the composite aerogels.

**Fig. 10:**
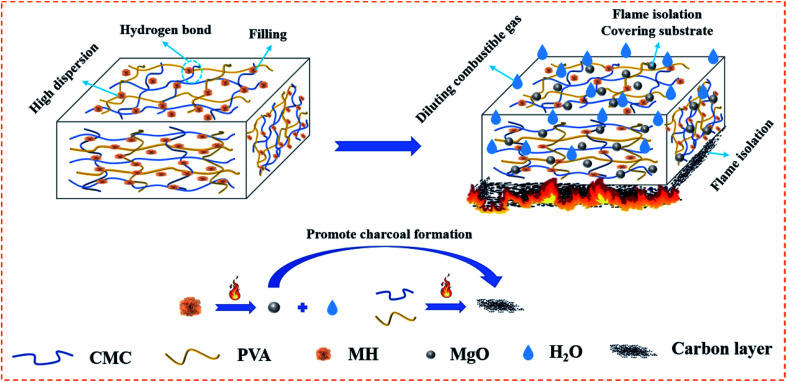
Enhancement mechanism of *in situ* growth of MH on CMC/PVA aerogels.

Several sets of composite aerogel samples were continuously exposed to the external flame of an alcohol lamp for up to 3 s to investigate the effect of MH on the combustion behaviour of CMC/PVA. The original CP aerogel was ignited within 2 s of contact with the flame source (Fig. 9a), and the limiting oxygen index (LOI) was only 22.4%. However, after the addition of MH, the polymer aerogel showed excellent capability to slow down the flame propagation; the LOI value of CP/MH60 reached 35.5% which is higher than other reported results at the same loading of MH.^53^ This finding shows that MH obtained *via in situ* formation with calcined magnesite as the raw material can greatly improve the flame retardant efficiency of MH in the polymer aerogel matrix, which can be attributed to the flower cluster morphology of MH and its high dispersion in the polymer matrix. The flame retardancy of MH applied to other polymeric substrates is given in Table 2 for reference. Fig. 10 shows the flame retardant mechanism of MH on CMC/PVA aerogel. On the one hand, MH decomposition at high temperature produces water vapor which can absorb heat as well as dilute O_2_ around the combustible material, on the other hand, the decomposition produces MgO with high melting point which can cover the surface layer of the substrate and promote the denseness of the carbon layer as well as form a barrier between the flame and the substrate.

**Table 2:** Flame retardant parameters of MH/polymer

Sample	MH concentrations (%)	LOI (%)
MH/PP^53^	60	32.8
MH/asphalt^54^	25	25.3
PE/EVA/MH^55^	55	25
EVA/MH^56^	50	23.9
EVA/MH^56^	55	26.8

## 4. Conclusions

A simple hydration method was used to generate MH directly in CMC/PVA mixed sol with active MgO obtained by calcined magnesite as the raw material. The freeze-drying method was utilised to prepare a series of CMC/PVA/MH composite aerogels. SEM showed the successful *in situ* generation of nanoflower clusters of MH with good dispersibility in the porous network of the CMC/PVA aerogel. In addition, the *in situ* formation of MH improved the flame retardant efficiency of MH in the polymer matrix. When the MH content was 60%, the LOI value of the polymer aerogel reached 35.5%. The mechanical properties, water resistance and thermal stability of the composite aerogels were also considerably improved.

## Author contributions

Xiaohu Qiang: supervision, project administration, review & editing. Xin Guo: writing – original draft, investigation, formal analysis. Hongxi Su: investigation. Hong Zhao: investigation. Chengwei Ouyang: validation. Dajian Huang: conceptualization, supervision, writing – review & editing, funding acquisition.

## Conflicts of interest

The authors declare that they have no known competing financial interests or personal relationships that could have appeared to influence the work reported in this paper.

## Supplementary Material
